# Detection of imported COVID-19 cases worldwide: early assessment of airport entry screening, 24 January until 17 February 2020

**DOI:** 10.1186/s41182-020-00260-5

**Published:** 2020-09-14

**Authors:** Varvara A. Mouchtouri, Zacharoula Bogogiannidou, Martin Dirksen-Fischer, Sotirios Tsiodras, Christos Hadjichristodoulou

**Affiliations:** 1 Laboratory of Hygiene and Epidemiology, Faculty of Medicine, University of Thessaly, 22, Papakyriazi street, Larisa, Greece; 2 EU Healthy Gateways Joint Action, Larissa, Greece; 3 Hamburg Port Health Center, Hamburg, Germany; 4 National and Kapodistrian University of Athens Medical School, Athens, Greece

**Keywords:** COVID-19, Entry screening, Airports, Airplane, Traveler, SARS-CoV-2

## Abstract

The purpose of this study was to provide an overview of entry screening measures applied at airports in response to the COVID-19 epidemic worldwide. Between 24 January and 17 February 2020, 5.2% (95% CI 3.1–8.5) of the 271 total imported COVID-19 cases worldwide (excluding imported cases arriving in China, Macao, and Hong Kong) with known detection location were captured through airport entry screening. The majority of imported COVID-19 cases (210) were identified by the health care system (77.5%). Efforts should focus on health care system preparedness for early case detection, since according to our and previous studies health care facilities are the actual point of entry of imported cases.

## Introduction

Airport entry screening measures were implemented in various countries worldwide in response to severe acute respiratory syndrome (SARS), influenza pandemic (H1N1), and Ebola virus disease in West Africa, and detected no cases or a low number of cases [1]. However, several important secondary positive effects of entry screening have been reported including discouraging travel of ill persons, raising awareness, educating the traveling public, and maintaining operation of flights from/to the affected areas [1].

In response to the novel coronavirus disease 2019 (COVID-19) public health emergency of international concern, exit screening was implemented in affected areas, while many countries worldwide implemented entry screening at international airports in an attempt to identify imported cases. Entry screening aims at assessing the presence of symptoms and/or the exposure to COVID-19 of travelers arriving from affected areas, and travelers that have been identified as exposed to or infected with COVID-19 should be quarantined or isolated and treated [1].

The purpose of this study was to provide an overview of entry screening measures applied at airports during the first days of the COVID-19 epidemic worldwide (excluding imported cases arriving in China, Hong Kong, and Macao). On 13 January 2020, Thailand detected the first COVID-19 case outside Wuhan, China [2]. We collected information about the countries implementing entry screening from 24 January until 17 February 2020, the number of travelers that were identified outside China as positive for COVID-19 at airports (primary cases) and their secondary cases, and how many imported cases (primary cases and secondary cases) were identified in community settings including health care, quarantine facilities, and others.

## Methods

To identify articles and reports containing information about airport entry screening data for countries worldwide excluding imported cases arriving in China, Hong Kong, and Macao, we searched grey literature including the situation reports from the World Health Organization (WHO), the technical reports from the European Centre for Disease Prevention and Control (ECDC), and online newspapers. We used the Google web search engine to identify articles and reports published from 24 January until 17 February 2020, using the terms “airport”, “screening” “coronavirus”, “border check”, and “health check”.

Data were extracted from 255 WHO and ECDC reports and newspaper articles. Publications were reviewed in order to extract data about the number of imported COVID-19 cases that were detected by countries worldwide (excluding imported cases arriving in China, Hong Kong, and Macao) through: (a) entry screening at airports (primary cases and their secondary cases), (b) the health care system (primary cases and their secondary cases), and (c) quarantine implemented after repatriation of nationals from affected areas.

Proportions of the cases detected according to the detection location and type of country (implementing or not implementing airport entry screening) and 95% confidence intervals (CI) were calculated using normal approximation of the binomial model. Statistics were performed using Microsoft Excel.

## Results

From 24 January until 17 February 2020, 26 countries reported 362 imported cases (Table 1 in the Appendix). Eighteen out of 26 countries conducted entry screening at airports. Five countries (Germany, Belgium, Finland, Spain, and Sweden) did not implement entry screening at airports during the period of our study while three other countries (Sri Lanka, Cambodia, and Nepal) initiated screening following the confirmation of the first imported case. However, they did not report another imported case until 17 February 2020. These eight countries reported 24 cases in total. A total of 271 cases had a known detection location and were reported in countries that implemented airport entry screening measures during the study period.

Our study showed that 14 out of 271 total imported cases with known detection location in countries which conducted entry screening [5.2%, 95% confidence intervals (CI) 3.1–8.5] were captured through entry screening at airports, and this proportion increased to 9.2%, 25 out of 271 (95% CI 6.3–13.3) when adding the 11 cases detected after conducting public health observation to persons detected through entry screening (Table 2 in the Appendix). A total of 15 secondary cases were detected through contact tracing of the primary cases detected by entry screening (5.5%, 95% CI 3.4–8.9). In these countries which conducted entry screening as presented in Table 2 in the Appendix, 21 cases were captured through quarantine among repatriated travelers (7.7%, 95% CI 5.1–11.6). The majority of imported cases (210) in countries which implemented entry screening at airports were identified by the health care system (77.5%, 95% CI 72.1–82). The numbers of imported cases detected at airports and in community settings worldwide until 17 February 2020 are presented in Table 2 in the Appendix.

## Discussion

Modeling work conducted by ECDC for COVID-19 demonstrated that approximately 75% of cases from affected Chinese cities would arrive at their destination during the incubation period and remain undetected, even if the efficacy of the screening test to detect symptomatic individuals was 80% for both exit and entry screening [3]. Another modeling study estimated that 9% of imported cases could be detected through entry screening (95% CI 2–16) if exit screening was being implemented (44% would be identified through exit screening), and the remaining 46% (95% CI 36–58) would be undetected [4].

Travelers with mild symptoms, asymptomatic, pre-symptomatic, or those concealing symptoms (with antipyretics) cannot be detected through entry screening and will enter a country [5]. Our study confirmed conclusions from health measures taken at borders during previous epidemics, indicating that the de facto point of entry into the healthcare system for travelers with serious infectious diseases was found to be the in-country, acute care facilities (hospitals, clinics) and not the airports [6].

Appraisal of airport entry screening measures have shown that it is highly resource demanding [7]. In addition, investing in entry screening at airports might decrease resources from other important response measures including preparedness of the health care system, information campaigns to travelers and the communities, and stockpiles of medical supplies. Moreover, entry screening may give the public a false sense of security. However, decision-making about entry screening implementation should take into consideration the particular outbreak characteristics globally and at the country level, as well as the country’s priorities, epidemiological profile, and financial issues.

Our study is limited since the results are based on grey literature. Another limitation is that for a large proportion of cases (67, 19%), a location of detection could not be determined. Moreover, we did not appraise entry screening measure protocols implemented in each country. Entry screening may have applied different protocols, e.g., fever screening and/or exposure assessment, temperature measuring devices, and thresholds, and screening effect may have differed from country to country. We can assume that implementation of exit screening measures at the departing airports of the affected countries reduced the numbers of affected travelers arriving who were detected through entry screening, but to our knowledge there are currently no publications reporting exit screening data for our study period.

Our study demonstrated that a small proportion 14.8%, 40/271 (95% CI 11.0–19.5), of imported COVID-19 cases can be detected through entry screening and related activities in countries which implemented entry screening. Some countries may consider entry screening worthwhile even for detecting this small number of imported cases, especially during the early stage of the epidemic, in order to gain time and coordinate the public health response [8]. However, decisions should be taken after considering the entry screening limitations, the effectiveness of other measures such as quarantine of travelers arriving from high risk areas, and the available resources [1, 9, 10].

## Conclusions

Only a very small proportion of cases were detected at airports during entry screening. Entry screening alone cannot be effective to prevent importation of cases but could be considered as a supplementary response measure to information strategies at the airports and to preparedness at hospitals. If airport entry screening is considered to supplement response measures, the impact and opportunity costs for other areas of the response such as community mitigation and hospital response should be carefully weighed. The health system should be prepared to detect the imported cases and to prevent nosocomial COVID-19 infections [6]. During the early phase of the epidemic, public health authorities should be prepared to identify contacts early and to prevent further spread to the community.

Lessons learned from entry screening during the early “delay phase” of the epidemic could also be useful when considering the measures to be taken as part of the mitigation phase, where exit/entry screening measures could be combined with pre-travel screening, molecular testing and/or antibody testing for SARS-CoV-2 [10].

## Data Availability

The datasets used to draw the conclusions are provided in this letter. Conclusions drawn in this letter reflect authors’ views.

## Appendix

**Table 1:** Number of COVID-19 imported cases per region according to the detection location in the country (*N* = 362)

**Region**	**Airport screening (*****N*** **= 40)**			**Health care settings (*****N*** **= 231)**		**Cases among repatriated travelers who were detected in quarantine facilities**	**Cases with unknown /uncertain place of detection**	**Total**
	**Primary cases**	**Primary cases detected during public health observation initiated after screening**^**a**^	**Secondary cases**	**Primary cases**	**Secondary cases**^**b**^			
**Western Pacific**	11	4	6	65	96	14	47	243
**South East Asia**	3	3	4	5	10	2	12	39
**Americas**	0	0	0	13	3	3	4	23
**European**	0	3	5	12	23	4	0	47
**Eastern Mediterranean**	0	1	0	1	3	1	4	10
**Total,** ***N*** **= 362 (%)**	14 (3.9)	11 (3.0)	15 (4.1)	96 (26.5)	135 (37.3)	24 (6.7)	67 (18.5)	362 (100)

Source: Grey literature including online newspapers and governmental reports searched from 24 January to 17 February 2020

^a^Primary cases detected during public health observation initiated after screening: cases among the passengers who were asymptomatic when they passed the airport screening but they were put under public health observation and they developed symptoms during the time they were under observation

^b^Secondary cases at health care settings: the number of secondary cases including those identified through contact tracing of the primary cases and during quarantine of contacts of the primary cases

**Table 2:** Imported cases of COVID-19 detected at airports and in the community worldwide (*N* = 362)

**Region**	**Country**	**Number of cases (primary and secondary) according to the detection setting**			**Unknown**	**Total imported cases**
		**Airport entry screening**	**Health care system**	**Quarantine of possibly exposed incoming travelers**		
Western Pacific	Japan	0	28	8	23	59
	Singapore	5	58	4	10	77
	Malaysia	5	15	2	-	22
	Vietnam	0	4	0	12	16
	Taiwan	6	14	0	-	20
	Cambodia^a^	N/A	1	0	-	1
	Philippines	0	3	0	-	3
	Australia	0	15	0	-	15
	South Korea	4	23	1	2	30
South East Asia	Sri Lanka^a^	N/A	1	0	-	1
	Nepal^a^	N/A	1	0	-	1
	India	3	0	0	-	3
	Thailand	7	13	2	12	34
Americas	Canada	0	8	0	-	8
	USA	0	8	3	4	15
Eastern Mediterranean	United Arabic Emirates	1	4	0	4	9
	Egypt	1	0	0	-	1
European	France	0	12	0	-	12
	Germany^b^	N/A	14	2	-	16
	UK	6	3	0	-	9
	Italy	0	2	1	-	3
	Russian Federation	2	0	0	-	2
	Belgium^b^	N/A	0	1	-	1
	Finland^b^	N/A	1	0	-	1
	Spain^b^	N/A	2	0	-	2
	Sweden^b^	N/A	1	0	-	1
Total number of cases in countries implementing entry screening at the time the cases were detected, *N* = 271^C^ (%)		40 (14.8)	210 (77.5)	21 (7.7)	N/A	N/A
Total, *N* = 362 (%)		40 (11)	231 (63.8)	24 (6.7)	67 (18.5)	362 (100)

^a^Country that initiated entry screening at airports after identifying the first imported case

^b^Country that did not implement entry screening at airports

^c^Total number of cases with known detection location in countries that implemented entry screening at the time the cases were detected

## Abbreviations

COVID-19: Coronavirus disease 2019
WHO: World Health Organization
ECDC: European Centre for Disease Prevention and Control
CI: Confidence intervals
SARS-CoV-2: Severe acute respiratory syndrome coronavirus 2
